# Meta-Analysis of Risk Factors and Incidence of Interstitial Pneumonia With CHOP-Like Regimens for Non-Hodgkin Lymphoma

**DOI:** 10.3389/fonc.2022.880144

**Published:** 2022-06-01

**Authors:** Jing Yang, Limin Chai, Junting Jia, Liping Su, Zhiying Hao

**Affiliations:** ^1^Department of Pharmacy, Shanxi Province Cancer Hospital/Shanxi Hospital Affiliated to Cancer Hospital, Chinese Academy of Medical Sciences/Cancer Hospital Affiliated to Shanxi Medical University, Shanxi, China; ^2^Department of Haematology-Oncology, Shanxi Province Cancer Hospital/Shanxi Hospital Affiliated to Cancer Hospital, Chinese Academy of Medical Sciences/Cancer Hospital Affiliated to Shanxi Medical University, Shanxi, China

**Keywords:** CHOP-like chemotherapy, IP, NHL, risk factor, incidence

## Abstract

**Objectives:**

Interstitial pneumonitis (IP), a potentially fatal complication of non-Hodgkin Lymphoma (NHL) patients received CHOP (cyclophosphamide and doxorubicin and vincristine and prednisone)-like chemotherapy, negatively affected patients’ clinical outcome and quality of life. We aimed to explore patient-related, disease-related and drug-related risk factors associated with IP and gain a better understanding of the incidence in NHL patients.

**Methods:**

Databases, including PubMed, Ovid, China National Knowledge Internet (CNKI), and Wanfang Database from inception to January 20, 2022, were searched to identify studies evaluating the risk factors and incidence of IP. The included studies were assessed by Newcastle-Ottawa Quality Scale and above 7 points was considered high quality. The statistical analysis of risk factors was assessed by RevMan software (version 5.3) and incidence of IP was calculated by R software (version 4.1.2). Fixed-or random-effects models were applied to estimated the relative risks (RRs) and 95% confidence interval (Cl).

**Results:**

A total of 12 studies comprised of 3423 NHL patients were included in the analysis. Among the 3 available patient-related risk factors, 6 disease-related risk factors and 3 drug-related risk factors, it was found that only drug-related risk factors were significantly associated with IP development: pegylated liposomes doxorubicin (PLD) replacement (RR = 3.25, 95% CI = 1.69-6.27, *I^2 =^* 64%), rituximab (RTX) addition (RR = 4.24, 95% CI = 2.58-6.96, *I^2 =^* 0) and granulocyte colony stimulating factor (G-CSF) administration (RR = 5.80, 95% CI = 3.05-11.05, *I^2 =^* 0). The pooled incidence of CHOP, R-CHOP, and R-CDOP regimen was 1.0% (95% CI 0.00-0.01, *I^2^* = 8%), 7.0% (95% CI 0.05-0.09, *I^2^* = 64%) and 22.0% (95% CI 0.13-0.32, *I^2^* = 87%) respectively.

**Conclusion:**

PLD replacement, RTX addition and G-CSF administration were significant risk factors of IP for NHL patients received the CHOP-like chemotherapy. Clinicians should focus on these patients to detect and treat the IP development timely, which might bring benefit in patients’ survival.

**Systematic Review Registration:**

PROSPERO, identifier CRD42022309884.

## 1 Introduction

Non-Hodgkin lymphoma (NHL) has shown a gradually increase since the last century in both developed and developing countries (1–3). The cyclophosphamide, doxorubicin, vincristine, and prednisone (CHOP) regimen has become a standard chemotherapy for NHL for more than 40 years. The complete-response rate and progression free survival, as well as overall survival in NHL patients have improved significantly since the new antitumor drugs was introduced into clinical practice. However, with the extensive use of CHOP-like regimens (CHOP, R-CHOP, R-CDOP), it was observed more and more patients who were treated with CHOP-like regimens experienced adverse events in interstitial pneumonitis (IP). IP is a lethal complication in NHL patients undergoing chemotherapy, characterized with diffuse pulmonary interstitial infiltrates in early stage and pulmonary interstitial fibrosis in late stage (4). IP may cause dyspnoea, respiratory failure, and death (5). The therapeutic efficacy may compromise in patients who experienced IP due to delay and premature termination of chemotherapy.

The analysis of incidence and risk factors of IP were inconsistent among different hematology centers worldwide. Based on reported literatures, the incidence of IP in NHL patients ranged widely from 0.03% to 29.0% (6, 7). A number of clinical trials showed great differences of risk factors, including patient-related risk factors, (e.g., gender, age and smoking habit), disease-related risk factors, [e.g., histology, Ann Arbor stage, IPI scores, levels of dehydrogenase (LDH), levels of β2-microglobulin (β2-MG), and B symptom], drug-related risk factors, [rituximab (RTX), pegylated liposomal doxorubicin (PLD), and granulocyte colony stimulating factor (G-CSF)] and so much more (8–18). CHOP-like regimens are the standard and the widest used chemotherapy for the treatment of NHL while the exact conclusion of risk factors for CHOP-like regimens related to IP in patients with NHL are lacking. Therefore, this meta-analysis was performed to evaluate risk factors and the incidence of CHOP-like regimens induced IP in patients who received chemotherapy. This information can help clinicians assess the risks and benefits when prescribing CHOP-like regimen for NHL patients.

## 2 Methods

### 2.1 Literature Search

We carried out a systematic literature search to collect all of the potential eligible studies about IP in patients with NHL, from which we selected observational studies and randomized control trials (RCTs). Three authors independently searched for electronic databases for articles published up to 20 January 2022, including PubMed, Ovid, China National Knowledge Internet (CNKI), and WanFang Database. The search terms were ‘[(cyclophosphamide and doxorubicin and vincristine and prednisone) or (CHOP)] and (interstitial pneumonia)’ in all fields in English and Chinese versions. This search was further supplemented by screening the references of the retrieved articles and review articles so as not to miss any correlated studies. Subsequently, each article was checked according to our inclusion criteria.

### 2.2 Inclusion and exclusion criteria

The inclusion criteria were as follows: (1) prospective and retrospective clinical studies; (2) studies including patients diagnosed with NHL, regardless of subtypes; (3) patients enrolled in the study received CHOP-like regimen; (4) data about either the incidence of IP or risk factors for CHOP-like regimen; The exclusion criteria were as follows: (1) number of cases (enrolled patients) less than 10 patients; (2) significant differences in the baseline characteristics; (3) non-classical (3-weekly) CHOP-like chemotherapy; (4) conference abstract, review, comment, case report, studies that reported in-complete information, and cell or animal study.

### 2.3 Data Extraction

Three researchers independently screened the articles and reached an agreement on all items. The following data were recorded from the included articles: (a) study characteristics (first author, year of publication, country, study design, lymphoma subtypes, chemotherapy regimen), (b) demographic characteristics (age, gender, number of patients, number of patients eligible for IP, risk factors and incidence of IP).

### 2.4 Clinical Endpoint

The primary outcome of this meta-analysis were risk factors associated with IP and the incidence of IP among patients with NHL undergoing CHOP-like treatment.

### 2.5 Quality Assessment

Included studies were assessed by Newcastle-Ottawa Quality Scale. The scale form had a total of 9 points and assigned a maximum of 4 points for subject choice, 2 points for comparability between groups, 3 points for exposure or outcome. 7 points indicated high-quality.

### 2.6 Statistical Analysis

The events or incidences of IP were extracted from the data of all included studies. The incidence of IP for each article was calculated as the number of patients who experienced IP divided by the total number of patients along with corresponding exact 95% confidence interval (CI). The pooled incidence of overall IP was estimated using the meta package in R software (version 4.1.2; R Foundation for Statistical Computing, Beijing, China), and risk factors associated with IP were done using RevMan software (version 5.3). The pooled incidence of IP was estimated using both fixed-effects model and random-effects model and presented pooled weighted results as relative ratios (RRs) with both 95% CIs and 95% prediction intervals. Cochran *Q* test and *I^2^* statistics were applied to assessed the extent of statistical heterogeneity across studies, and *I^2^* larger than 50 was defined as heterogeneity. Fixed effects model was used for low heterogeneity of the pooled results; Otherwise, a random effect model was used. Sensitivity analysis was performed after removing one study every time to examine its impact on the results of the meta-analysis.

## 3 Results

### 3.1 Literature Search and Characteristics

**Figure 1:**
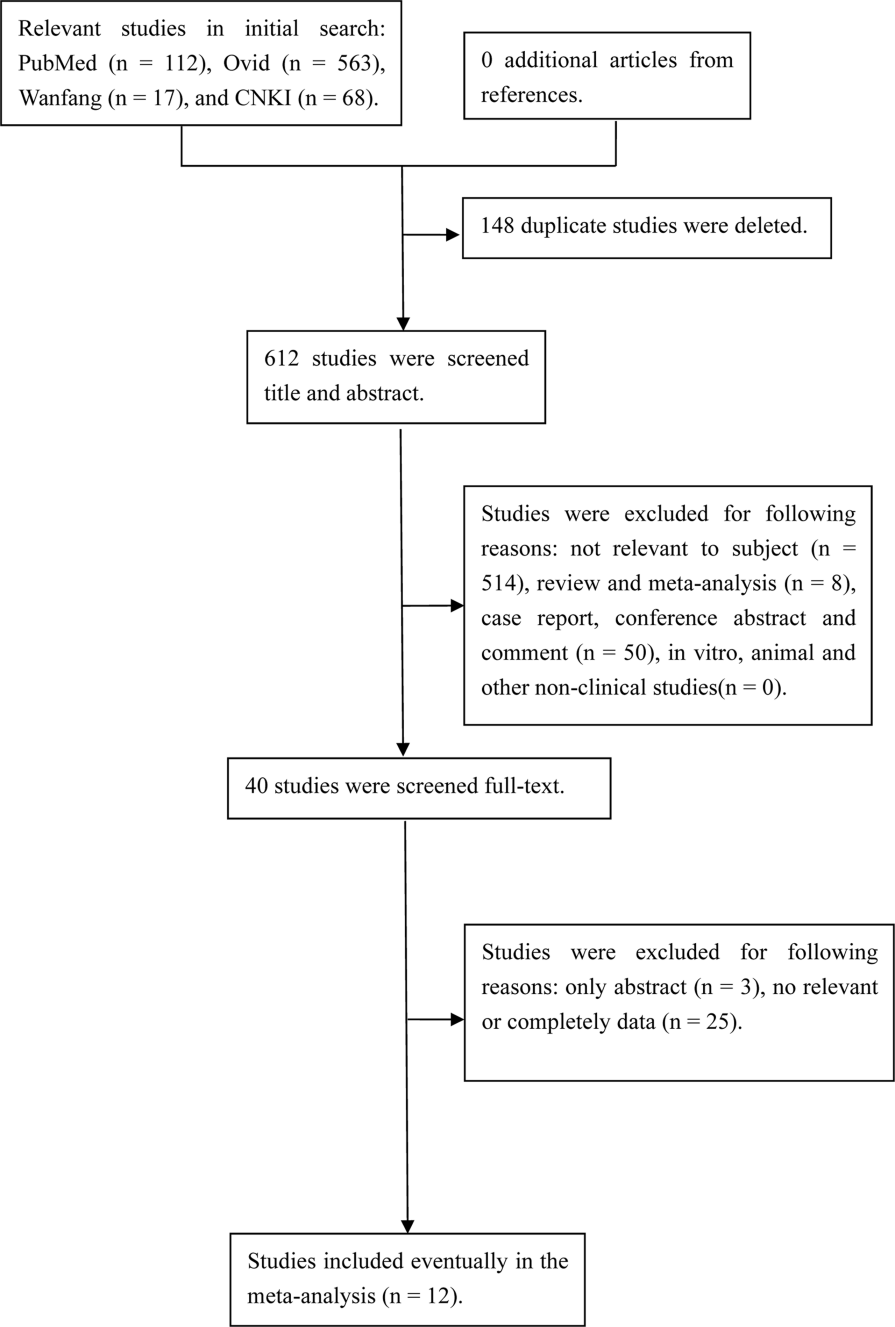
Flow diagram of the literature search.

The initial search yielded 479 relevant references. After removing duplicate studies and then screening title and abstract of remaining studies, 32 articles were further assessed at full-text level. Finally, 12 prospective studies with 3423 exposures were included in the analysis. The study selection process is shown in **Figure 1**. The characteristics of the studies are listed in **Table 1**.

**Table 1:** The characteristic of included studies.

study	country	histology	study design	Risk factors	Incidence of IP			
					CHOP (N=), n (%)	CHOP(N=), n (%)	R-CDOP (N=), n (%)	Quality scores
Ennishi (14)	Japan	B-cell NHL	Retrospective, single-center	gender, stage, RTX	105 (0)	90 (14.4)	–	7
Huang (15)	Taiwan, China	DLBCL	Retrospective, single-center	age, gender, stage, IPI,LDH, RTX	267 (1.9)	262 (8.0)	–	9
Katsuya (9)	USA	B-cell NHL	Retrospective, single-center	gender, stage, IPI, RTX, G-CSF	59 (1.7)	129 (6.2)	–	7
Kurokawa (17)	Japan	B-cell NHL	Retrospective, single-center	gender, stage, RTX	121 (0)	114 (4.4)	–	7
Lim (16)	Korea	NHL	Retrospective, single-center	gender, IPI, RTX	29 (3.5)	71 (7.0)	–	7
Meng (7)	China	DLBCL	Retrospective, single-center	age, gender, stage, LDH, β2-MG, PLD	–	114 (2.6)	114 (29.0)	8
Pan (18)	China	DLBCL	Retrospective, single-center	age, gender, stage, LDH, β2-MG, B symptom, PLD	–	72 (15.3)	73 (31.5)	9
Salmasi (13)	Canada	B-cell NHL	Retrospective, single-center	RTX	231 (1.3)	329 (4.0)	–	7
Wang (11)	China	B-cell NHL	Retrospective, single-center	age, gender, stage, LDH, β2-MG, B symptom, RTX	42 (2.4)	61 (14.8)	–	8
Wei (8)	China	DLBCL	Retrospective, single-center	age, stage, IPI, LDH, RTX, PLD,G-CSF,	186 (1.1)	191 (5.2)	179 (8.4)	8
Zheng (10)	China	B-cell NHL	Retrospective, single-center	gender, stage, RTX, PLD	102 (2.0)	180 (7.8)	95 (23.2)	8
Zhou (12)	China	B-cell NHL	Retrospective, single-center	gender, stage, RTX, PLD	89 (0)	57 (1.8)	38 (21.1)	8

NHL, non-Hodgkin lymphoma; DLBCL, diffuse large B cell lymphoma; IP, interstitial pneumonitis; LDH, dehydrogenase; β_2_-MG, levels of β_2_-microglobulin; RTX, rituximab; PLD, pegylatedliposomal doxorubicin; G-CSF, granulocyte colony stimulating factor

### 3.2 Quality Assessment

The included observational studies met most of the quality assessment criteria, and total score of all these studies was greater than 7, indicating all of high quality. The Newcastle-Ottawa Scale are also shown in **Table 1**.

### 3.3 Meta-Analysis of Risk Factors

#### 3.3.1 Patient-Related Risk Factors

**Figure 2:**
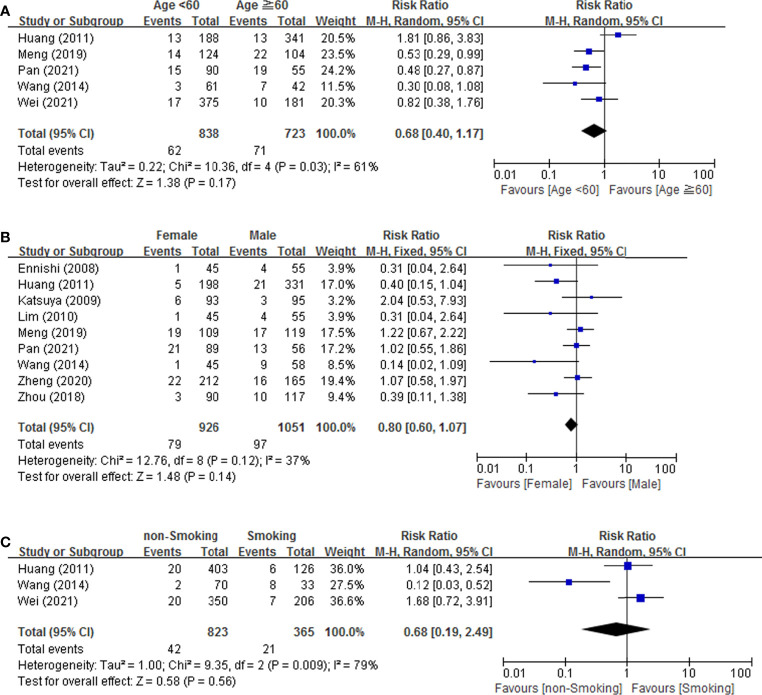
Forest plot of studies among patient-related risk factors associated with IP development. **(A)** Age. **(B)** Gender. **(C)** Smoking.

Meta-analysis conducted for patient-related risk factors including age, gender and smoking habit are shown in **Figure 2**. The result indicated that age (RR = 0.68, 95% CI = 0.40-1.17), gender (RR = 0.80, 95% CI = 0.60-1.07), and smoking habits (RR = 0.68, 95% CI = 0.19-2.49) was not associated with the risk of IP development. We found that age (*p* = 0.03, *I^2^* = 61%) and smoking habits (p = 0.009, *I^2^* = 79%) showed moderate heterogeneity in meta-analysis. No heterogeneity was found for gender (*p* = 0.12, *I^2^* = 37%).

#### 3.3.2 Disease-Related Risk Factors

**Figure 3:**
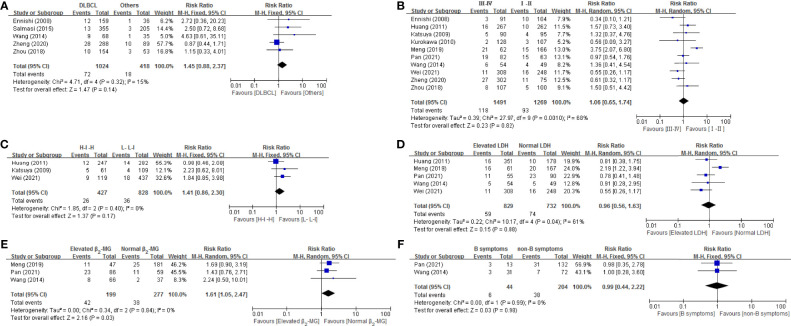
Forest plot of studies among disease-related risk factors associated with IP development. **(A)** Histology. **(B)** Stage. **(C)** IPI. **(D)** LDH. **(E)** β2-MG. **(F)** B symptom.

As shown in **Figure 3**, 6 disease related risk factors (histology, Ann Arbor stage, IPI score, LDH, β_2_-MG and B symptom) were all performed for a meta-analysis. The result revealed that none of the 6 risk factors, histology [(RR = 1.45, 95% CI = 0.88-2.37), Ann Arbor stage (RR = 1.06, 95% CI = 0.65-1.74), IPI score [(0-2, L-L-I), (3-5, H-I-H)] (RR = 1.41, 95% CI = 0.86-2.3), LDH (RR = 0.96, 95% CI = 0.56-1.63), β_2_-MG (RR = 1.61, 95% CI = 1.05-2.47) and B symptom (RR = 0.99, 95% CI = 0.44-2.22)], significantly increased the risk. Four of these factors, histology (*p* = 0.32, *I^2^* = 15%), IPI score (*p* = 0.40, *I^2^* = 0), β_2_-MG (*p* = 0.84, *I^2^* = 0) and B symptom (*p* = 0.99, *I^2^* = 0%), had no heterogeneity and the remaining two factors, Ann Arbor stage (*p* = 0.001, *I^2^* = 68%) and LDH (*p* = 0.04, *I^2^* = 61%) had significant heterogeneity.

#### 3.3.3 Drug-Related Risk Factors

**Figure 4:**
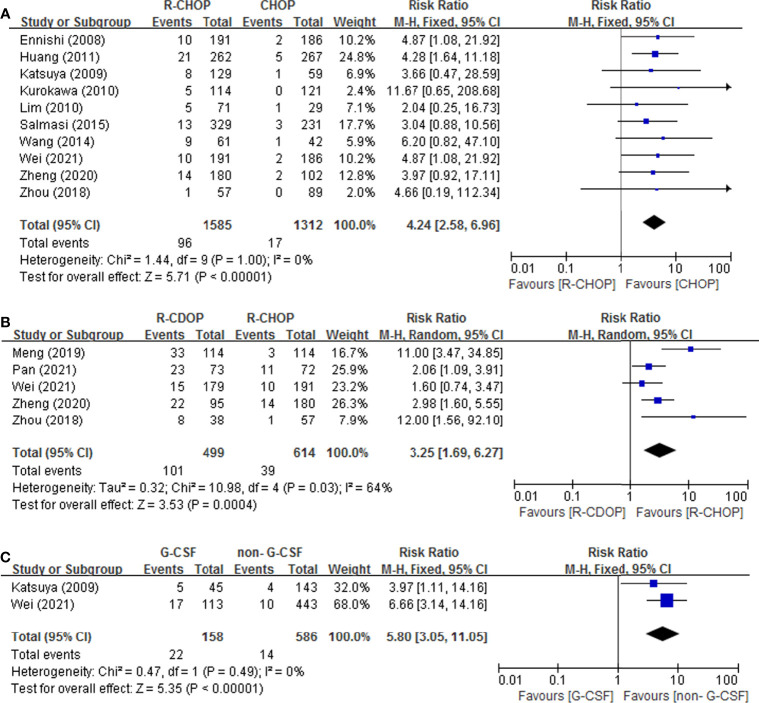
Forest plot of studies among drug-related risk factors associated with IP development. **(A)** RTX. **(B)** PLD. **(C)** G-CSF.

In **Figure 4**, all drug-related risk factors available for meta-analysis were performed on PLD replacement (yes vs. no), RTX addition (yes vs. no), and G-CSF administration (yes vs. no). Our results indicated that PLD replacement (RR = 3.25, 95% CI = 1.69-6.27), RTX addition (RR = 4.24, 95% CI = 2.58-6.96) and G-CSF administration (RR = 5.80, 95% CI = 3.05-11.05) were all significantly associated with the risk of IP in lymphoma patients. There were no heterogeneity for RTX addition and G-CSF administration. PLD replacement (*p* = 0.03, *I^2^* = 64%) presented significant heterogeneity.

### 3.4 Meta-Analysis of Incidence

#### 3.4.1 Incidences of IP in CHOP-Like Regimens

**Figure 5:**
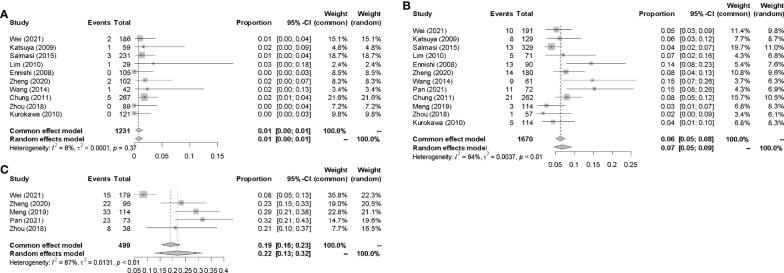
Incidences of IP in CHOP-like regimens. **(A)** CHOP. **(B)** R-CHOP. **(C)** R-CDOP.

Data for the events or incidences of IP were available for pooled analysis from a total of 1231, 1670, 499 patients eligible in 10, 12, 5 prospective clinical studies which were assigned CHOP, R-CHOP, and R-CDOP therapy, respectively. The highest incidence of IP was 31.5% in R-CDOP group (7), and the lowest incidence of IP was 0 in R-CHOP group (14). The pooled IP incidences were 1.0% (95% CI 0.00-0.01, *I^2^* = 8%), 7.0% (95% CI 0.05-0.09, *I^2^* = 64%) and 22.0% (95% CI 0.13-0.32, *I^2^* = 87%) in CHOP, R-CHOP and R-CDOP group respectively (**Figure 5**).

#### 3.4.2 Incidences of IP in G-CSF Administration Group

**Figure 6:**
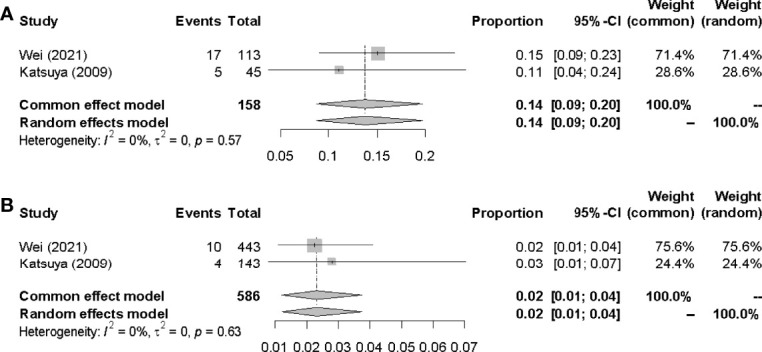
Incidences of IP in G-CSF administration group. **(A)** G-CSF group. **(B)** non-G-CSF group.

The pooled incidences of IP in G-CSF administration group and non-G-CSF administration group were 14.0% (95% CI 0.09-0.20, *I^2 =^* 0%) and 2.0% (95% CI 0.01-0.04, *I^2^* = 0%) respectively(**Figure 6**).

#### 3.4.3 Incidences of IP in Easterners and Westerners

**Figure 7:**
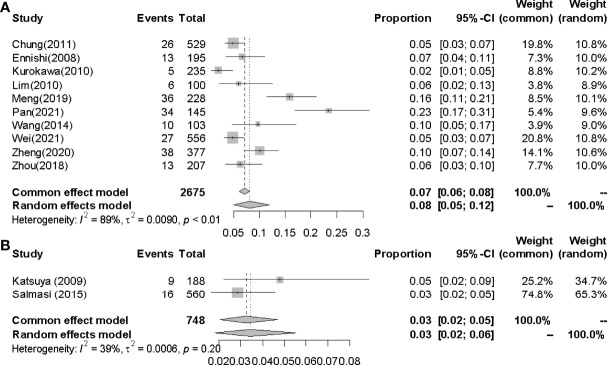
Incidences of IP in Easterners and Westerners. **(A)** Easterners. **(B)** Westerners.

The pooled incidences of IP in Easterners and Westerners were 8.0% (95% CI 0.05-0.12, *I^2^* = 89%) and 3.0% (95% CI 0.02-0.05, *I*^2^ = 39%) respectively (**Figure 7**).

## 4 Discussion

This study aimed to identify the risk factors associated with IP so that clinicians could early assess severe pulmonary adverse effects and adjust treatment decisions in time. The results suggested that drugs-related factors were related to incidence of IP, while patient-related risk factors (age, gender, smoking habit) and disease-related factors (histology, stage, IPI score, levels of LDH, levels of β2-MG and B symptom) were not. The pool incidences of IP were 1.0%, 7.0%, and 22.0%, in CHOP, R-CHOP, and R-CDOP groups respectively.

Among the patient-related risk factors and disease-related factors analyzed, significant heterogeneity was observed in age (*I^2^* = 61%), smoking habit (*I^2^* = 79%), Ann Arbor stage (*I^2^* = 68%), and levels of LDH (*I^2^* = 61%). For age, no significant heterogeneity existed after the trial from Huang (15) (*p* = 0.54, *I^2^* = 0%) removed. Although heterogeneity existed, Huang believed that age was not a risk factor, as there was no significant difference regarding to age ≥60 years with IP (50.0% vs 65.2%, *p* = 0.196) in his study, which was consistent with other four articles (7, 8, 11, 18). For smoking habit, the opinion of the Wang (11) was conflicting with the other two studies (7, 15), proving results of the heterogeneous (p = 0.56, *I^2^* = 79%). Wang considered that the history of cigarette smoking was a factor associated with IP, while the latter hold a contrary view. The total number of patients included in Wang’s article was 103, while in the other two articles was more than 500. The heterogeneity might be owed to the small number of patients included in Wang’s study. The heterogeneity of disease stage and levels of LDH were all derived from the different conclusion between Meng (7) and others. The results of Meng indicated that disease stage and elevated LDH were associated with high incidence of IP, however, others took an opposite opinion. Meng’s conclusion that disease stage and elevated LDH were risk factors may be confused with the use of high-dose PLD as there were more stage III-IV patients in the high-dose PLD group. This heterogeneity might be attributed to the high dose of PLD, which was identified as a risk factor in all reports.

Recently, an increasing number of case reports about additional and more concerning RTX-related IP have been described (19–24). The pooled incidence of IP in CHOP therapy increased from 1.0% to 7.0% following the addition of RTX to CHOP. RTX-induced IP may be associated with imbalance metabolism with B cell and T lymphocyte, the cytotoxic substances induction and release (25, 26). In addition, RTX can bring B-cell depletion for a long time, increasing the risk of opportunistic infection (27).

In this meta-analysis, R-CDOP group has a higher pooled incidence compared with R-CHOP group [7.0% (95% CI 0.05-0.09, *I^2^* = 67%) vs 22.0% (95% CI 0.13-0.32, *I^2^* = 87%)]. The results of sensitivity analysis showed that the source of heterogeneity was Wei’s data (8). In R-CDOP groups, the IP incidences were 8.4% according to Wei’s report, while others were all higher than 20% (28.9%, 23.1%, 21.1%, 31.5%) (7, 10, 12, 18). To investigate the cause of incidence heterogeneity between R-CDOP group, a subgroup analysis should be performed. However, due to the lack of detailed data of patients with IP in the RCDOP group, subgroup analysis was not conducted. There may be some risk factors associated with the incidence of IP in R-CDOP group, which need further investigation. Given the baseline demographic, study design and definition of IP were consistent with the other four studies, so we added its result into the pooled incidence calculation.

Several studies of patients with hematological malignancy experienced PLD-associated severe pulmonary toxicity. PLD combination chemotherapy for patients with Hodgkin’s lymphoma and relapsed/refractory multiple myeloma, 16.7% (5/30) and 12.5% (1/8) patients experienced >grades 3 serious pulmonary toxicity (28, 29). As the dose of PLD elevated from 25-30 mg/m^2^ to 35-40mg/m^2^, the incidence of IP in DLBCL patients accordingly increased from 17.30% to 38.71% (p <0.05) (7). Clinicians should pay attention to this fatal adverse effect and choose PLD carefully. The exact mechanism of high incidence of IP induced by PLD is still unknown, but it seems to be due to the direct damage to lung and remain longer in circulation due to the polyethylene glycol coat.

It was observed a higher pooled rate of IP in patients receiving G-CSF compared with patients who did not receive G-CSF (14% vs 2%). Apart from chemotherapeutic drugs, G-CSF was considered as another risk factor correlated with the incidence of IP. A few studies reported that IP could develop in patients with various hematological malignancies, particularly NHL, especially at the recovery phase of chemotherapy-induced leucopenia during G-CSF administration (30). When received G-CSF after administration of pneumotoxic anti-cancer agents, such as bleomycin, G-CSF could accelerate the number and activity of neutrophils, which may cause an aggravation of bleomycin pneumonia (30–33). The impact of G-CSF on pulmonary toxicity had previously been suggested by others. G-CSF increased the number of neutrophils, stimulated proliferation and activation of alveolar macrophages, and surged the production of proinflammatory cytokines, all of which may be involved in the pathogenesis of IP (34, 35).

The results of this meta-analysis showed Easterner patients had slightly higher incidence of IP than Western patients (8.00% vs 3.00%). The events of PLD-related IP were nearly all occurred in Easterner as R-CDOP scheme are mainly adopted in China, which might lead the higher rate. There was some concern that Asian patients may have a genetic predisposition to drug-induced IP (13, 36), and it was possible that race may accounted for the different pervalence of IP.

This was the first meta-analysis to summarize available data about risk factors for IP, finally it was identified that three drug-related risk factors with convincing evidence, which could provide clear evidence for clinical practice. Several shortcomings of our analysis were worth to be considered. Firstly, racial differences existed in the included population, which may lead to publication bias. Secondly, due to various disease-related and treatment-related factors in the patients enrolled in the clinical studies, it was hard to analyze all the confounding factors because of lacking of detailed information from the included studies. In addition, a limited number of studies were included, especially for some risk factors (G-CSF therapy, smoking, IPI scores and levels of β2-MG). In the literatures we searched, there was no report regarding the impact of how drugs were infused-the time of infusions on the pulmonary complications, which should be also considered for as it might affect the occurrence of adverse reaction. Although IP is a serious complication, it has been reported that clinical course can resolved after the treatment with sulfanilamide and glucocorticoids. At present, the treatment in the literature is only empirical, and further clinical research is needed.

## 5 Conclusion

In conclusion, IP should be highlighted in NHL patients who received CHOP-like regimen containing RTX and PLD, especially in those who received G-CSF for chemotherapy-induced leucopenia. In any CHOP-like treated patient who complained with respiratory symptoms, the complication of IP should be considered and appropriate investigations requested quickly. Early recognizing and effectively management of drug-related pulmonary toxicity are important for NHL patients. Further prospective studies with multicenter large sample are required to verify our viewpoint.

## Data Availability Statement

The original contributions presented in the study are included in the article/supplementary material. Further inquiries can be directed to the corresponding authors.

## Author Contributions

JY, LC and JJ designed the study and drafted the manuscript. ZH and LS edited the manuscript and were involved in the conception of the study. All authors approved the manuscript and agreed to be accountable for all aspects of the research.

## Conflict of Interest

The authors declare that the research was conducted in the absence of any commercial or financial relationships that could be construed as a potential conflict of interest.

## Publisher’s Note

All claims expressed in this article are solely those of the authors and do not necessarily represent those of their affiliated organizations, or those of the publisher, the editors and the reviewers. Any product that may be evaluated in this article, or claim that may be made by its manufacturer, is not guaranteed or endorsed by the publisher.
